# Health care utilisation among older people with Down syndrome compared to specific medical guidelines for health surveillance: a Swedish national register study

**DOI:** 10.1186/s12913-020-05800-7

**Published:** 2020-10-15

**Authors:** G. Ahlström, A. Axmon, M. Sandberg, E. Flygare Wallén

**Affiliations:** 1 Department of Health Sciences, Faculty of Medicine, Lund University, Box 157, 221 00 Huddinge, Sweden; 2 EPI@LUND (Epidemiology, Population studies, and Infrastructures at Lund University), Lund University, 221 00 Stockholm, Sweden; 3 Department of Neurobiology, Care Sciences and Society (NVS), H1 Division of Family Medicine and Primary Care, Karolinska Institutet (KI), Alfred Nobels allé 10, 141 83 Huddinge, Sweden; 4 Academic Primary Health Care Center, Solnavägen 1e, 113 65 Stockholm, Sweden

**Keywords:** Aged, Comorbidity, Down syndrome, Intellectual disability, Guideline adherence, Mental retardation, Specialist health care, Inpatients, Outpatient care

## Abstract

**Background:**

Specific medical guidelines for health surveillance exist for people with Down syndrome (DS) since 25 years but knowledge of adherence to the guidelines is lacking. The guidelines were developed to avoid unnecessary suffering from preventable conditions. The aims of the study were to investigate 1) planned health care visits in relation to the co-morbidities described in specific medical guidelines as a measure of adherence, 2) unplanned health care visits as a measure of potentially unmet health care needs and 3) gender differences in health care utilisation among older people with DS.

**Methods:**

This register-based study includes people with DS (*n* = 472) from a Swedish national cohort of people with intellectual disability (*n* = 7936), aged 55 years or more, and with at least one support according to the disability law, in 2012. Data on inpatient and outpatient specialist health care utilisation were collected from the National Patient Register for 2002–2012.

**Results:**

A total of 3854 inpatient and outpatient specialist health care visits were recorded during the 11 years, of which 54.6% (*n* = 2103) were planned, 44.0% (*n* = 1695) unplanned and 1.4% (*n* = 56) lacked information. More than half of the visits, 67.0% (*n* = 2582) were outpatient health care thus inpatient 33% (*n* = 1272). Most planned visits (29.4%, *n* = 618) were to an ophthalmology clinic, and most unplanned visits to an internal medicine clinic (36.6%, *n* = 621). The most common cause for planned visits was cataract, found at least once for 32.8% in this cohort, followed by arthrosis (8.9%), epilepsy (8.9%) and dementia (6.6%). Pneumonia, pain, fractures and epilepsy each accounted for at least one unplanned visit for approximately one-fourth of the population (27.1, 26.9, 26.3 and 19.7% respectively). Men and women had similar numbers of unplanned visits. However, women were more likely to have visits for epilepsy or fractures, and men more likely for pneumonia.

**Conclusions:**

Increased awareness of existing specific medical guidelines for people with DS is vital for preventive measures. The relatively few planned health care visits according to the medical guidelines together with a high number of unplanned visits caused by conditions which potentially can be prevented suggest a need of improved adherence to medical guidelines.

## Background

Longevity has increased dramatically in people with Down syndrome (DS) during the last decades. The main explanation is the progress in infant heart surgery, which has resulted in a decrease in mortality among children younger than one year of age from 40.8% in 1973 to 4.8% in 2003 [1]. However, living longer have consequences for age-related morbidity, with implications for health care and health surveillance for people with DS. The development of co-morbidities in the fourth to sixth decades of life is reported to be more prevalent among people with DS than in the population in general [2]. Thus, people with DS are more likely to need specialist care during their last years of life. Compared to the general population, many age-related changes in health and functional status, such as vision and hearing impairment, Alzheimer’s disease, thyroid disorders and epilepsy occur at an earlier age among people with DS [3]. Visual and hearing impairment is common among people with DS already in early ages [4], and increases sharply after 40 years of age [2]. In fact, hearing impairment may increase to the extent that up to 100% experiences hearing loss at the age of 60 [5]. Epilepsy has been reported to occur among 40% before one year of age and among an additional 40% in older people with DS in connection with Alzheimer’s disease [6]. Moreover, both Swedish and international population based studies have reported epilepsy to be a more frequent cause of death among people with DS than in the general population [7–9]. Additionally, epilepsy has been suggested to be the most prevalent cause for in-hospital mortality among people with DS [7].

The increased longevity for people with DS combined with the changed living context from institutions to living in the community, mostly in supportive housing, has changed the conditions for health care access for this population in Sweden. In the past, medical care was mainly provided in the institutions, whereas today, Swedish health policies state that people with DS should seek health care when needed in the same way as the general population. However, despite the special needs of people with DS, physicians with specialist education on adults or older people with DS are lacking, which, among other things, may jeopardise healthy ageing [2, 10].

National health care guidelines or recommendations for health surveillance of co-morbidities of DS, including planned health care visits, have been developed in many countries, for children as well as for adults [4, 11–13]. Regular health checks, which have been introduced in several countries, may be one strategy to address the barriers that people with DS might encounter in seeking health care [14]. In Sweden, even though guidelines exist for adults with DS since 1991 and for children with DS since 1985 [4, 11], there are no medical guidelines specific to older people that account for age-related diseases.

There have been reports of poor compliance with existing medical guidelines for people with DS [15, 16]. For example, although US guidelines state that obstructive sleep apnoea, atlanto-axial instability, and hearing or vision loss should be evaluated regularly in health care, less than 50% of adults with DS had been evaluated for any of these conditions during an 8.5-year period [15]. In the UK, one-third of a cohort of adults with DS had not had any medical assessment during the previous three years [17]. Even if Swedish specific medical guidelines for health surveillance of people with DS have existed for 25 years, adherence to the guidelines have not yet been investigated [1].

During the last decade, a few studies have focused on gender differences in people with DS with respect to morbidity [18], health care utilisation [19] and mortality [9]. Gender differences have been reported for epilepsy as a comorbid condition with dementia, being more prevalent in women with DS than in men with DS [18]. Furthermore, it is well-known that osteoporosis, and osteoporotic fractures are more common among older women than men in the general population [20]. This risk is even higher among women with DS as gonadal dysfunction, low hormone levels and early onset of menopause is more prevalent [21, 22]. Regarding hospitalisation, men with DS have been reported to have longer inpatient stays than women with DS [19]. The gender differences in disease pattern and health care utilisation motivate the development of gender-specific health surveillance for older people with DS.

Improving health surveillance is vital to enable healthy ageing in the older population with DS [23]. Health surveillance is reasonably planned health care beforehand, and adherence may be assessed by investigating planned health care visits in specialist care as many conditions require specialist knowledge to complement primary health care given by the general practitioner (GP). We previously reported that unplanned health care in somatic care exceeded planned visits for older people with intellectual disabilities with and without DS, and that older people with intellectual disabilities had fewer planned health care visits than the general population [24]. Studying health care utilisation as a method to investigate adherence to medical guidelines among older people with DS has not yet been performed. Therefore, health care utilisation patterns for people with DS in Sweden are not yet known, nor their connection to specific medical guidelines or reasons for unplanned visits. Taking into account that in Sweden, as in many other countries, there are no national registers of all forms of health care, we have used two reliable registers in this study that include all outpatient and inpatient specialist care and used them as proxy for health care utilisation. In fact, a national register for primary care is not available for this study. The aims of the present study were to investigate 1) planned health care visits in relation to the co-morbidities described in specific medical guidelines for people with DS as a measure of adherence to these guidelines, 2) unplanned health care visits as a measure of potentially unmet health care needs and 3) gender differences in health care utilisation among older people with DS.

## Methods

The present study is register-based, using Swedish national registers to establish the study cohort as well as identify health care utilisation.

### Setting

#### Health care

Health care in Sweden is funded by taxes. Mostly public but also private alternatives are available. The Health and Medical Services Act [25] regulates access to health care on equal terms for the entire population, based on an assessment of the needs of the individual. The first level of health care is primary health care. In order to receive second level care, i.e. specialist care, the patient usually needs a referral from the GP. For chronic conditions that requires specialist competence, the initial referral goes from the GP to a specialist clinic, but the subsequent contact between the patient and the specialist clinic is continued without any link to the GP. However, primary health care can keep the medical responsibility for general uncomplicated conditions [26]. People with DS, or their families, or staff in social service have themselves to initiate visits to the GP for an examination of a health problem. There are no formalised specialist health care services for intellectual disability or for DS.

#### Swedish registers used in the study

The Swedish intellectual disability services is based on the Swedish act concerning support and services for persons with certain functional impairments (Swedish abbreviation LSS) [27]. This act regulates measures of support and services for people with intellectual disability (including DS) and/or autism spectrum disorders. It is a right-based law that entitles adult people to apply for any number of 8 specified services. All support and service provided according to the LSS act is recorded in a register (LSS register).

The National Patient Register (NPR) includes records for all visits made in inpatient and outpatient specialist health care. For each visit, the register includes one primary and up to 21 secondary diagnoses determined by the responsible physician at discharge from hospital or outpatient visits, and coded according to the International Statistical Classification of Diseases and Related Health Problems 10th Revision (ICD-10).

Both the LSS register and the NPR are based on mandatory registration and are maintained by the Swedish National Board of Health and Welfare, a public authority with commissions from the government.

### Study cohort

All people who had received at least one form of LSS support and service during 2012 and who were alive and at least 55 years old at the end of that year (*n* = 7936) were included in the original cohort. For this study, we selected those who had at least one diagnosis of DS (ICD-10 code Q90) recorded in the NPR during 2002–2012.

Thus, the study cohort in the present study included 472 older people with DS, 247 men (52%) and 225 women (48%). Their mean age at the start of the 11-years study period, i.e. 1 January 2002, was 49.7 years (range 44–75). The majority of those included (*n* = 438, 93%) had a diagnosis of unspecified DS (Q90.9).

### Outcomes

Data on health care utilisation and diagnoses were collected from the NPR for the period 2002–2012. Primary diagnoses (i.e. the main causes for the health care contacts) were considered for planned health care visits in relation to co-morbidities as a measure of guideline adherence and unplanned visits as a measure of potentially unmet health care needs.

We specifically investigated the presence of known co-morbidities listed in the Swedish specific medical guidelines together with the more recently published guidelines for Norway [11–13] (Appendix 1).

Diagnoses were assessed on ICD-10 block level and as single diagnoses. For planned health care utilisation, we also investigated the following disease groups based on present medical guidelines for people with DS: arthrosis in different parts of the body (M15-M19), atlanto-axial instability (S13.4), cataract (H25-H26), coeliac disease (K90), constipation (K59), dementia (F00, F03.9, G30), depression (F32-F33), diabetes (E10-E14), epilepsy (G40-G41, R56.8), gastro-oesophageal reflux (K21), hearing loss (H90-H91), heart and lung symptoms (I20, I52), impacted cerumen (H61.2), keratoconus (H18.6), obesity (E65-E66), osteoporosis (M80-M81), pneumonia (J12-J15, J18, J69.0), sleep apnoea (G47.3), testicular cancer (C62), thyroid disorder (E03.9) and skin disease (L00-L99). For unplanned visits, we investigated epilepsy or seizure (G40-G41, R56.8), fractures in different parts of the body (S02-S92), pain in different parts of the body (M79.6, R07, R10, R52) and pneumonia (J12-J15, J18, J69.0).

### Statistics

Descriptive data on diagnoses and diagnostic groups (as described above) are presented for planned and unplanned visits separately, for the total study cohort as well as stratified by sex. For each diagnosis, we present 1) the number of people with this diagnosis as the primary diagnosis (i.e. cause for the visit) at least once during the study period and 2) the number of visits with this diagnosis as the primary diagnosis. Only diagnoses recorded for at least 10 people are included.

Gender differences were investigated with respect to a) number of visits, using the Mann-Whitney U test, as the data were skewed, and b) having at least one visit during each year, using generalised linear models (GLMs) with calendar year indicating repeated measures, estimating relative risks (RRs) with 95% confidence intervals (CIs). All analyses were performed in IBM Statistics SPSS 23.0. *P*-values below 0.05 were considered statistically significant.

## Results

### Health care characteristics

All 472 individuals had at least one registration recorded in inpatient or outpatient specialist health care during the study period, namely, the visit by which we identified the person as having DS. DS was recorded as the primary diagnosis, i.e., the main cause for the planned visit, at least once for 22.9% (*n* = 108) of the individuals, whereas intellectual disability (ICD-10 code F70-F79) was the primary diagnosis at least once for 11.7% (*n* = 55). For unplanned visits, DS was recorded as the main cause for the visit at least once for 7.0% (*n* = 33) and intellectual disability for 1.9% (*n* = 9) of the individuals (*n* = 472).

Regarding health care visits, totally 3854 registered visits were identified for the 472 individuals included. Of these, 54.6% (*n* = 2103) were planned, 44.0% (*n* = 1695) were unplanned, and 1.4% (*n* = 56) lacked information for these two types of visits. More than half of the visits, 67.0% (*n* = 2582) were outpatient health care thus inpatient 33% (*n* = 1272). Inpatient care visits represented 9.9% (*n* = 209) of the planned visits and 64.5% (*n* = 1094) of the unplanned visits. All 56 visits that lacked information on whether they were planned or unplanned were made in outpatient specialist care. The most frequently visited department was the internal medicine department, to which 791 visits (20.5%) were made (Table 1). Of these, 38.7% were outpatient health care visit, and 19.8% were planned. Inpatient care in the internal medicine department were 61.3, and 78.5% were unplanned visit.

**Table 1:** Health care visits for 472 older adults with Down syndrome during 11 years study period at the ten most visited clinics (total and different types of health care visits)

	Total people with DS *N* = 472	Total health care visits *n* = 3854	Planned health care visits *n* = 2103	Unplanned health care visits *n* = 1695	Unknown health care visits *n* = 56	Outpatient health care visits *n* = 2582	Inpatient health care visits *n* = 1272
	*n* (%)	*n* (%)	*n* (%)	*n* (%)	*n* (%)	*n* (%)	*n* (%)
Internal medicine	259 (54.9)	791 (20.5)	157 (7.5)	621 (36.6)	13 (23.2)	306 (11.9)	485 (38.1)
Ophthalmology	212 (44.9)	664 (17.2)	618 (29.4)	33 (1.9)	13 (23.2)	613 (23.7)	51 (4.0)
Surgery	170 (36.0)	484 (12.6)	194 (9.2)	284 (16.8)	6 (10.7)	285 (11.0)	199 (15.6)
Orthopaedic clinic	157 (33.3)	467 (12.1)	274 (13.0)	187 (11.0)	6 (10.7)	337 (13.1)	130 (10.2)
General psychiatric clinic	50 (10.6)	197 (5.1)	130 (6.2)	57 (3.4)	10 (17.9)	166 (6.4)	31 (2.4)
Ear, nose and throat clinic	80 (16.9)	191 (5.0)	139 (6.6)	50 (2.9)	2 (3.6)	158 (6.1)	33 (2.6)
Emergency department	79 (16.7)	168 (4.4)	8 (0.4)	160 (9.4)	0 (0)	152 (5.9)	16 (1.3)
Infectious diseases	68 (14.4)	114 (3.0)	12 (0.6)	102 (6.0)	0 (0)	18 (0.7)	96 (7.5)
Geriatrics and geropsychiatrics	61 (12.9)	158 (4.1)	140 (6.6)	17 (1.0)	1 (1.8)	136 (5.3)	22 (1.7)
Neurological clinic	40 (8.5)	93 (2.4)	69 (3.3)	24 (1.4)	0 (0)	67 (2.6)	26 (2.0)

Note: If multiple visits are recorded, only one visit per person, clinic and day is included

### Planned visits – adherence to specific medical guidelines

On the ICD-10 block level, the most common cause for a planned visit was disorders of lens (H25-H28), followed by chromosomal abnormalities, not elsewhere classified (Q90-Q99; Appendix 2). When assessing specific diagnosis (single ICD-10 codes), (Appendix 2), the most common cause for a planned visit for individuals in this cohort was senile cataract, unspecified (H25.9, 21.0%, *n* = 99, with 190 visits), followed by epilepsy, unspecified (G40.9, 6.0%, *n* = 30, with 66 visits).

When aggregating diagnoses according to known problems described in the specific medical guidelines (Table 2), the most common cause for a planned visit was cataract, followed by epilepsy, arthrosis and dementia.

**Table 2:** Primary diagnoses listed in specific medical guidelines, present for planned and unplanned visits for 472 older adults with Down syndrome (225 women and 247 men) with at least one visit in health care

	Planned				Unplanned			
	*n* (%)	Median	Q1-Q3	Min-max	*n* (%)	Median	Q1-Q3	Min-max
Arthrosis (M15-M19)								
Total	42 (8.9)	2	1–2	1–5	12 (2.5)	1	1–1	1–2
Women	30 (13.3)	2	1–2	1–5	9 (4.0)	1	1–1	1–2
Men	12 (4.9)	1	1–4	1–5	3 (1.2)	1	1–1	1–1
Cataract (H25-H26)								
Total	155 (32.)	1	1–2	1–3	8 (1.7)	1	1–1	1–1
Women	90 (40.0)	1	1–2	1–3	5 (2.2)	1	1–1	1–1
Men	65 (26.3)	1	1–2	1–3	3 (1.2)	1	1–1	1–1
Constipation (K59)								
Total	11 (2.3)	1	1–1	1–3	22 (4.7)	1	1–2	1–7
Women	6 (2.7)	1	1–1	1–1	11 (4.9)	1	1–2	1–7
Men	5 (2.0)	1	1–2	1–3	11 (4.5)	1	1–1	1–3
Dementia (F00, F03.9, G30)								
Total	31 (6.6)	1	1–2	1–4				
Women	20 (8.9)	1	1–2	1–3				
Men	11 (4.5)	1	1–2	1–4				
Diabetes mellitus (E10-E14)								
Total	15 (3.2)	1	1–3	1–11				
Women	8 (3.6)	1	1–2	1–11				
Men	7 (2.8)	2	1–3	1–3				
Epilepsy or seizure (G40-G41, R56.8)								
Total	42 (8.9)	1	1–2	1–6	93 (19.7)	1	1–2	1–30
Women	32 (14.2)	1	1–2	1–6	62 (27.6)	1	1–2	1–30
Men	10 (4.1)	1	1–2	1–3	31 (12.6)	1	1–1	1–4
Hearing loss (H90-H91)								
Total	10 (2.1)	1	1–1	1–2				
Women	3 (1.3)	1	1–1	1–1				
Men	7 (2.8)	1	1–1	1–2				
Impacted cerumen (H61.2)								
Total	19 (4.0)	1	1–1	1–5				
Women	9 (4.0)	1	1–1	1–2				
Men	10 (4.0)	1	1–1	1–5				
Keratoconus (H18.6)								
Total	11 (2.3)	1	1–2	1–2				
Women	4 (1.8)	1	1–2	1–2				
Men	7 (2.8)	1	1–2	1–2				
Pneumonia (J12-J15, J18, J69.0)								
Total					128 (27.1)	1	1–1	1–5
Women					46 (20.4)	1	1–1	1–5
Men					82 (33.2)	1	1–2	1–4

Note: Only diagnoses recorded for at least 10 people (planned and unplanned separately) are included. Excluded from the table due to too few observations: atlanto-axial instability (S13.4), coeliac disease (K90), depression (F32-F33), gastro-oesophageal reflux (K21), heart and lung symptoms (I20-I52), obesity (E65-E66), osteoporosis (M80-M81), sleep apnoea (G47.3), testicular cancer (C62), thyroid disorders (E039), and skin disease (L00-L99)

### Unplanned visits – potentially unmet health care needs

The most common cause for an unplanned visit, on the ICD-10 block level, was influenza and pneumonia (J09-J18), followed by general symptoms and signs (R50-R69; Appendix 2).

For the aggregated diagnoses described above, pneumonia, pain and fractures each corresponded to at least one unplanned visit for more than one-fourth of the study cohort (Table 3). A fracture of femur was the most common type of fracture (S72, 11.2%, *n* = 53, with 62 visits); the most common type of pain was abdominal pain (R10, 13.6%, *n* = 64, with 143 visits).

**Table 3:** Number and percentage of the four most frequent primary diagnosis (main causes) recorded for an unplanned visit, and its occurrence in planned visits for older adults with Down syndrome (total *n* = 472, 225 women and 247 men)

	Planned				Unplanned			
	*n* (%)	Median	Q1-Q3	Min-Max	*n* (%)	Median	Q1-Q3	Min-Max
Pneumonia (J12–15, J18, J69.0)								
Total					128 (27.1)	1	1–2	1–5
Women					46 (20.4)	1	1–2	1–5
Men					82 (33.2)	1	1–2	1–4
Epilepsy or seizure (G40-G41, R56.8)								
Total	42 (8.9)	1	1–2	1–6	93 (19.7)	1	1–2	1–30
Women	32 (14.2)	1	1–2	1–6	62 (27.6)	1	1–2	1–30
Men	10 (4.1)	1	1–2	1–3	31 (12.6)	1	1–1	1–4
Pain (M79.6, R07, R10, R52)								
Total	31 (6.6)	1	1–1	1–4	127 (26.9)	1	1–2	1–35
Women	20 (8.9)	1	1–1	1–4	66 (29.3)	1	1–2	1–35
Men	11 (4.5)	1	1–1	1–1	61 (24.7)	1	1–1	1–5
Fractures (S02-S92)								
Total	67 (14.2)	1	1–2	1–4	124 (26.3)	1	1–2	1–5
Women	44 (19.6)	1	1–2	1–4	72 (32.0)	1	1–2	1–5
Men	23 (9.3)	1	1–1	1–2	52 (21.1)	1	1–2	1–3

Note: Only diagnoses recorded for at least 10 people (planned and unplanned separately) are included

### Gender differences – diagnosis and type of health care visit

Regarding primary diagnosis (main cause) of the planned health care visit, more women than men have had at least one planned visit for cataract, dementia, epilepsy, and arthrosis (Table 2). Regarding the four most frequent primary diagnoses (Table 3), fractures accounted for at least one unplanned visit for almost one third of the women and about one fifth among men, and epilepsy, including seizure, accounted for at least one unplanned visit for one-fourth of the women and one eighth among men. In contrast, more men than women had had at least one unplanned visit for pneumonia (Table 3).

Regarding the number of total health care visits, there were no statistically significant differences between men and women (*p* = 0.200), men had a median of 5 visits (range 1–38) and women 6 (1–184). Men had fewer number of visits to specialist outpatient health care (*p* = 0.013), while the number of inpatient health care visits was similar. Also, whereas the number of unplanned visits was similar for men and women (*p* = 0.260), women had more planned visits than men (*p* = 0.002). A total of 17.4% men (*n* = 43) and 10.7% (*n* = 24) of women had no planned visits at all.

The regression analyses confirms that men were nearly as likely as women to have at least one yearly visit in outpatient specialist care (RR 0.96, 95% CI 0.91–1.02) and inpatient care (RR 1.07, 95% CI 0.97–1.18). Men were also equally likely to have at least one yearly unplanned visit (RR 1.06, 95% CI 0.97–1.15), but they were somewhat less likely to have at least one planned visit (RR 0.92, 95% CI 0.86–0.99).

## Discussion

More than half of the health care visits were planned (assessed as a proxy for adherence to specific medical guidelines) and almost half unplanned (assessed as proxy for unmet needs of preventive interventions) during the 11-year study period. The number of planned visit must be regarded as surprisingly few with respect to the existing specific medical guidelines. The most common causes for a planned visit were cataract, arthrosis, and epilepsy, and after that dementia, which is reasonable from the perspective of the existing medical guidelines and known co-morbidities. However, due to the observed time period and the few visits made at the individual level, health surveillance needs to be further evaluated for older people with DS, not at least from the perspective of an ageing population.

A large proportion of these older people with DS had at least one unplanned visit caused by fractures, pain, pneumonia and epilepsy. As these are well-known conditions, this could at least partly illustrate a lack of adherence to specific medical guidelines and preventive measures. If so, this finding is troublesome, given the extra burden for the person that an unplanned visit to a health care provider can entail especially in this group with decreased cognitive and communication ability [2]. Overall the health care utilisation was similar between men and women. However, men had fewer visits to outpatient health care visit and fewer planned visits than women. This result is not consistent with the few previous studies that have reported differences between men and women with DS using health care such as one study that report longer stay in hospitals for men compared with women with DS [19, 28] and slightly more hospital admissions [28]. However, the previous studies included people at younger ages and no outpatient data. Further research is needed before a conclusion can be drawn about possible differences in access as well as use of health care for men and women with DS, which is an issue in many countries.

### Planned visits – adherence to specific medical guidelines

Almost 20 % of the men and just over 10 % in total in our study had not had any planned visits at all during the 11 years. It is well-known that morbidity increases with age in DS more than it does in the general population [2, 5]. Thus this result is unexpected considering existing medical guidelines [11–13]. Australia has a similar health care system to Sweden in that primary health care and the GP are the first contact for the population for non-emergency health care [29]. Another study from Australia reports that people with ID are getting fewer referrals to specialist care compared to people without ID from their GPs in primary health care [30, 31]. Weise and colleagues identified from previous research several barriers of access to the health care system [29] for people with ID. These were an ill-equipped health workforce, care staff untrained to recognise signs of common physical and mental ill-health and therefore missed necessary subsequent actions, diagnostic difficulties including diagnostic overshadowing of ID and low health literacy among people with ID. To the best of our knowledge, there are no Swedish studies on this issue.

The most frequent cause for planned visits was age-related cataract, which occurred for about one-third of the individuals who had at least one planned visit. Specific medical guidelines recommend a visit to an eye health care specialist at least every fifth year for people with DS [11–13]. In addition, earlier reports have shown the prevalence of eye problems increasing with age [23] and reaching around 60% in older people with DS [2, 16]. Thus, the number of recorded visits due to cataract was far below what would have been expected if compliance to the specific medical guidelines. One possible reason for fewer visits may be that the responsibility within the health care system is not established for these follow-ups which for certain persons with communication difficulties, such as people with DS, might be crucial for visits to be made [2]. Decreased sight has many negative consequences in everyday life, and the prevalence of visual impairment increased with the severity of ID and with age [32]. Therefore, the older persons with DS need to be invited for a regular follow-up not least to the extent that the medical guidelines recommend.

There were few recorded planned visits for individuals in this study with a recorded primary diagnosis such as cervical spondylosis (CS) or heart problems [5]. Capone and colleagues report in a recently published systematic review an estimated prevalence of 60% of adults with DS that need health surveillance caused by cervical spondylosis and 35% caused by previously repaired or uncorrected congenital heart diseases (CHD) [5]. In the general population, the clinical presentation and manifestation of CS vary, and a safe diagnosis require multidimensional medical assessment [33]. Although CHDs among people with DS are well-known and currently well-treated at young ages, specific medical guidelines recommend that more attention should be given to the elevated risks of those with DS both with and without CHDs of developing cardiac morbidities later in life, such as mitral valve prolapse and pulmonary arterial hypertension [13, 34]. We have previously reported a higher prevalence of heart failure among people with ID (including people with DS) compared to the general population when including all secondary diagnosis from health care visits [35]. CHD and cervical spondylosis are common causes to specialist health care visits, and the few recorded visits in this study need to be further investigated. However one possible reason could be the high rate of early death and that people with DS in this study, were all alive at the end of the study period, which might be the healthiest ones as they have survived to this high age [9]. In our previous national study, we found that people 42 years and older with DS had 11 times higher mortality risk than a matched control population. They also died earlier compared with people with other intellectual disability diagnoses, with the mean age at death being 63.5 years in those with DS, compared with 72.1 years in those with other intellectual disability diagnoses and 76.2 years in the control population [9].

Orthopaedic problems such as arthritis are painful, and in the more recent specific medical guidelines from Norway yearly follow-up is recommended [13]. Only one-third of the study cohort had visited an orthopaedic clinic during the study period. We have previously reported that people with intellectual disabilities are less likely to have prescriptions for non-steroidal anti-inflammatory drugs (NSAIDs) [36, 37] and people with falls are more likely to be treated at other departments than orthopaedic as inpatients [38]. A Finnish population-based study reported that 20% of patients with DS in an age group over 30 years had had at least one orthopaedic surgery during their life, including fractures and dislocations [16]. Several publications have reported positive results with pain-relief and improved function from hip surgery in people with DS [39, 40]. Only a small number in this cohort had any reported orthopaedic surgery for joint implants which call for studies investigating potential needs for future improvements for this target group.

It is essential that planned visits for older people with DS includes the assessment of the risk of developing dementia. Previous studies have found the prevalence of dementia to be low before the age of 30 years and then increase to a prevalence of 70–80% for those over 65 years [2, 41]. The possible explanations of the discrepancy to the few registrations of dementia in this study is that we investigated only specialist health care and that the diagnoses are often made in primary health care. The relatively low amount of visits at neurological or in geriatrics and geropsychiatric clinics can explain the few registrations of dementia in this study.

Leaders at group homes and day program services in Sweden have expressed a wish for ageing people with intellectual disabilities to have the opportunity to see a physician at least once a year [42]. This has been introduced in several countries, and others have reported the need for such visits [13, 17, 43]. Johansson and colleagues [42] reported a lack of experience and competence among staff in detecting the need for assistive devices or increased care. Based on the time since the institutions were closed in Sweden and that the cohort in the present study comprises older people with DS it is reasonable to expect that the absolute majority lived in supported housing such as group homes during the study period. Within our Swedish national cohort of older people with intellectual disabilities, 76% of all people were supported in group homes in 2012 (*n* = 7936, unpublished data).

### Unplanned visits – potentially unmet health care needs

Unspecified abdominal pain was the most common pain diagnosis at unplanned health care visits. We have previously found that diagnoses of visceral pain and pain related to the urinary system were more common among people with intellectual disabilities than in the general population [37]. If the specific medical guidelines were updated with recommended regular screening for e.g. gastrointestinal disorders, treatment of pain might be initiated earlier and the number of unplanned health care visits may be reduced.

Almost one third of women in the study cohort had at least one fracture during the study period, with fractures of the femur being the most common type. The prevalence of fractures in the population with DS is far greater than that seen in the general population for the same age group and time period (just over 10%) [44]. To some extent, it may be caused by osteoporosis, which earlier research has found more prevalent especially in women with DS [5, 45] due to late menarche and early menopause [21]. In the population in general, the number of fractures increases with age, therefore the early ageing among people with DS could be one explanation for this higher prevalence of fractures. Osteoporosis is included in the more recent published specific medical guidelines from Norway for a regular follow-up [13] and the high level of fractures might be possible to reduce with a better adherence to the guidelines. We have reported on the need for fall prevention among older people with intellectual disability [38]. Falls occurred twice as often during vital activities among people with intellectual disabilities than in the general population. After a fall, people with intellectual disabilities most often experienced head or leg injuries and were more likely to require specialist care [38]. There is a need to further investigate if osteoporosis is being underdiagnosed, if relevant treatment is provided and if older people with DS are offered resources such as fall prevention similar to those recommended with high priority in national guidelines for the population in general [46]. A systematic risk assessment, investigation and treatment after the first fracture can reduce the proportion of new fractures with 40% [46].

In this study, unplanned visits were caused frequently by pneumonia and epilepsy, both well-known diseases among people with DS, especially in older age groups and in particular if dementia is present [47]. Both pneumonia and epilepsy have been reported to be more common as causes of death among people with DS than in the general population [1, 7, 9]. We have previously reported respiratory diseases as the main cause of death, accounting for 37% among people with DS [9]. This proportion rose to 50% when contributing causes were included. In addition, mortality from respiratory failure has been reported with a RR of 9.8 for in-hospital mortality among those with DS compared to the general population [7]. Respiratory diseases such as pneumonia are regarded as an ambulatory care sensitive condition (ACSC) [48] responsive to health care interventions, and thus such interventions may improve the health and quality of life in people with DS.

Although epilepsy was a common cause of both planned and unplanned health care visits, only 40 people (8.5%) had visited a neurological clinic during the study period. For those who have developed dementia, the prevalence of epilepsy is reported to be more than 80% [49, 50]. A previous study showed that despite medication, over half of those with epilepsy still reported experiencing seizures [51]. We have previously reported that epilepsy is the cause of death for a considerable number of people with DS [9]. Epilepsy, similarly to pneumonia, is considered an ACSC, thus unplanned hospitalisations should be possible to prevent [48]. Further studies should investigate whether older people with DS are treated in an optimal manner for epilepsy within primary health care and with adequate support from neurological specialist health care.

Seeking unplanned care could be a consequence of poorly followed specific medical guidelines, which has been reported in other countries [16, 17], or that older people with DS lack access to a primary health care provider who can adequately meet their complex needs [52]. Such difficulties may lead to delays in diagnoses, poorer disease management and unnecessary death [53]. Thus, regardless of diagnosis, the high number of unplanned visits, on which the results from the present study are consistent with our earlier report on people with intellectual disabilities in general [24], most likely indicates less optimal health care for an already vulnerable population. It is also unclear if it is the primary health care or the specialist health care that are responsible for the follow-ups according to guidelines comprising health surveillance of the ageing population with DS. This high number of unplanned visits cannot solely be explained by economic factors, as the health care in Sweden is mainly financed by taxes. Instead, we would like to propose two alternative explanations: 1) a lack of awareness in the health care system, as well as among supporting social service staff, of issues related to ageing adults with DS, which has been identified in interviews with managers and staff in intellectual disability services [42, 54, 55], and 2) difficulties in obtaining adequate health care for older people with DS and with, among other disabilities, communication difficulties [30].

DS was the recorded primary diagnosis of a visit to specialist health care at least once for almost one third of this cohort. This is earlier reported in studies on cause of death in this population [8, 9, 56]. The fact that a patient’s disability is recorded as the primary cause for a health care visit or cause of death might overshadow the actual reason for their health condition or cause for visit.

### Strength and limitations of the study

A major strength of this study is the use of the NPR, which is of high validity and has a 99% coverage rate of all somatic and psychiatric diagnoses registered at discharge [57]. It is mandatory for all health care providers, whether privately and publicly funded, to deliver data to the NPR, except for primary care [57]. Another strength of the study is that it examines a national sample of individuals with DS only, without the heterogeneity of studies of individuals with intellectual disability in general. However, people with no specialist health care visit at all under the 11 years period and thus without registration of DS are not included. However, we do not believe this affects the generalizability of the results.

The generalizability of the results from this study to people with DS in other countries is dependent both on differences within health care systems and in how people with disability are regarded in the country concerned. The Swedish health care and the social service system for people with intellectual disability is largely decentralised to community care, mostly financed by taxes and supposed to be delivered equally to everyone in the population based solely on need. We believe that our results may be applicable to other countries with similar conditions. However, the results might have limited generalizability to some low and middle-income countries with a limited system of specialist health care where people with DS meet additional problems such as stigma, and lack of confidence for authorities [58, 59]. Other obstacles for people with disability in low and middle-income countries are related to poverty with higher risks for morbidities and travelling to the hospital which is impossible for many people living in the countryside [60, 61].

This national sample only includes those with DS who had received services according the LSS law in 2012 and who had at least one visit in inpatient or outpatient specialist care in 2002–2012 (not including primary health care) during which a diagnosis of DS was recorded. Thus, we have failed to include those who live their lives without service and support from the municipality. However, it may be reasonable to believe that most older people with DS would have some kind of support and service according to LSS. Parents to older people with DS are not expected to have the ability to be caregivers due to own diseases or are not alive. Also, the oldest people in this study have grown up and lived the main part of their life in large institutions according to the disability policy that was in place before the 1980s [62]. In addition, the majority ought to have visited a health care provider at least once during these 11 years, especially with respect to age-related diseases. Thus, we believe that the majority of older people with DS are included in our cohort.

A possible weakness is that the individuals studied were all born during a time when the median age of people with DS was only 4 years [1]; thus this cohort consist of survivors, as all participants included were alive at 55 years of age and the survival age was nationally 63.5 years [9]. Therefore, it may be argued that these older individuals are the healthiest among those with DS. However, even if this may be the case, this cannot be a reason for failing health checks in accordance with specific medical guidelines in older people with DS according to the coherent research showing these people having more disease burden than in the general population [2, 9, 24].

We used specialist planned and unplanned health care on national data for evaluation of follow-up of medical guidelines developed with the goal to avoid unnecessary suffering from preventable conditions. The fact that the result is based only on specialist health care limits the generalizability to national results and must be taken into account when interpreting conclusions. However, the specialist health care is the best existing data as primary health care data not are registered at a national level in Sweden today. Many health conditions listed in the guidelines require examination in specialist health care. However, some diseases and problems listed in the specific medical guidelines, such as hypothyroidism and obesity, are probably followed up and examined in primary health care. The limitation that our data did not include common uncomplicated health conditions needs to be kept in mind when interpreting the result from this study. The scarce knowledge available highlight a need for representative studies of primary health care use in people with intellectual disabilities [31]. Future research also needs to identify potential inequalities caused by specific barriers for people with intellectual disabilities to access health care [63].

## Conclusions

Our data indicate deficiencies in adherence to specific medical guidelines and recommendations for health surveillance concerning people with DS. The low number of planned visits in relation to recommendations for specific disorders indicates few referrals to specialist health care from GPs or staff at specialist health care lacking awareness about the early ageing in the DS population. The high number of unplanned visits due to preventable conditions may represent potentially unmet health care needs within primary health care.

We suggest stronger efforts in implementing existing medical guidelines, updated for an older population in terms of fractures and pain in particular. DS is a population with extensive co-morbidity that now are ageing in a way that not existed earlier. Future research is warranted investigating prevention measure both within inpatient and outpatient specialist health care, as well as primary health care on a national level.

## Data Availability

In order to approve the study, the Regional Ethical Review Board in Lund made restrictions regarding access to the data due to the sensitive information on a very vulnerable group, i.e., persons with DS. Even though the data are anonymised, it contains enough details to enable identification of single individuals. Therefore, the datasets in the current study are available from the PI (GA) on reasonable request and only after approval from the Regional Ethical Review Board. However, as the database is compiled by national register data, other researchers may contact the register holders, the Swedish National Board of Health and Welfare and Statistics Sweden, to get access to the registries used in this study, and thereby generate a similar database.

## Appendix 1

**Table 4:** Medical guidelines of health checks for optimal medical care of adults with Down syndrome

	International [11]	Norway [13]	Sweden [12]
Alzheimer’s disease (dementia)	X	X	
Arthrosis		X	
Atlantoaxial Instability	X	X	Yearly
Autism/ADHD			X
Autoimmune disorders e.g., coeliac disease	X	X	X
Behavioural change	X	Yearly	
Blood screening		Yearly	Yearly
Cardiac and lung evaluation i.e., mitral valve prolapse, aortic regurgitation and pulmonary artery hypertension	X	X	Yearly
Contraceptive for females	X		
Cervical spine	X		Yearly
Dental care/oral health	X	Every 6 months	Yearly
Depression/anxiety	X	X	
Diabetes (blood glucose)		Yearly	
Emotional support	X		
Epilepsy, life periods with increased risk	3rd, 5th and 6th decade	X	
Eye examination i.e., cataract, refraction error, other eye disorders	Every 2 years	Yearly/ specialist every 5th year	Every three years until age 25
Gastrointestinal disorders i.e., dysphagia, oesophageal reflux, constipation, Hirschsprung’s disease		Yearly	Yearly
Hearing examination (hearing loss) conductive/sensorineural	Every 2 years	Yearly	Yearly
Infectious diseases such as pneumonia and influenza		Yearly	Yearly
National screenings			X
Nutrition	X		Yearly
Obesity	X	Yearly	
Orthopaedic problems (hips/knee/feet/spine)	X		Yearly
Osteoporosis		X	
Psychosocial motor and mental disorder	X		
Skin diseases i.e., alopecia, vitiligo, dermatitis, psoriasis		X	
Sleep, i.e., obstructive sleep apnoea syndrome			Yearly
Testicular cancer		Yearly	
Thyroid screening	Yearly	Yearly	Yearly

Note: X implies recommendation for follow-up whenever the person is in contact with health care

## Appendix 2

**Table 5:** Primary diagnoses on ICD code block level among 472 older adults (225 women and 247 men) with Down syndrome with at least one visit in planned and unplanned care. Only diagnoses within blocks recorded for at least 10 people are included (planned and unplanned separately)

	Planned				Unplanned			
	n (%)	Median	Q1-Q3	Min-Max	n (%)	Median	Q1-Q3	Min-Max
Other bacterial diseases (A30-A49)								
Total	1 (0.2)	1	1–1	1–1	25 (5.3)	1	1–1	1–4
Women					9 (4.0)	1	1–1	1–1
Men					16 (6.5)	1	1–1	1–4
Diabetes mellitus (E10-E14)								
Total	15 (3.2)	1	1–3	1–11				
Women	8 (3.6)	1	1–2	1–11				
Men	7 (2.8)	2	1–3	1–3				
Organic, including symptomatic, mental disorders (F00-F09)								
Total	23 (4.9)	1	1–1	1–2				
Women	14 (6.2)	1	1–1	1–2				
Men	9 (3.6)	1	1–1	1–2				
Mental retardation (F70-F79)								
Total	55 (11.7)	1	1–2	1–6				
Women	28 (12.4)	1	1–2	1–6				
Men	27 (10.9)	1	1–3	1–6				
Other degenerative diseases of the nervous system (G30-G32)								
Total	14 (3.0)	1	1–2	1–3				
Women	9 (4.0)	1	1–2	1–2				
Men	5 (2.0)	1	1–1	1–3				
Episodic and paroxysmal disorders (G40-G47)								
Total	43 (9.1)	1	1–2	1–6	61 (12.9)	1	1–2	1–30
Women	32 (14.2)	1	1–2	1–6	39 (17.3)	1	1–2	1–30
Men	11 (4.5)	1	1–2	1–3	22 (8.9)	1	1–1	1–4
Disorders of conjunctiva (H10-H13)								
Total	14 (3.0)	1	1–1	1–4				
Women	7 (3.1)	1	1–1	1–4				
Men	7 (2.8)	1	1–1	1–1				
Disorders of sclera, cornea, iris and ciliary body (H15-H22)								
Total	24 (5.1)	1	1–2	1–4				
Women	9 (4.0)	2	1–2	1–4				
Men	15 (6.1)	1	1–3	1–4				
Disorders of lens (H25-H28)								
Total	136 (28.8)	1	1–2	1–4				
Women	80 (35.6)	1	1–2	1–4				
Men	56 (22.7)	1	1–2	1–3				
Disorders of ocular muscles, binocular movement, accommo-dation and refraction (H49-H52)								
Total	19 (4.0)	1	1–1	1–3				
Women	13 (5.8)	1	1–1	1–2				
Men	6 (2.4)	1	1–1	1–3				
Diseases of external ear (H60-H62)								
Total	21 (4.4)	1	1–2	1–7				
Women	9 (4.0)	1	1–1	1–3				
Men	12 (4.9)	1	1–2	1–7				
Other disorders of ear (H90-H95)								
Total	10 (2.1)	1	1–1	1–2				
Women	3 (1.3)	1	1–1	1–1				
Men	7 (2.8)	1	1–1	1–2				
Ischaemic heart diseases (I20-I25)								
Total					11 (2.3)	1	1–1	1–3
Women					5 (2.2)	1	1–1	1–3
Men					6 (2.4)	1	1–1	1–2
Other forms of heart disease (I30-I52)								
Total	14 (3.0)	1	1–1	1–2	25 (5.3)	1	1–1	1–2
Women	6 (2.7)	1	1–2	1–2	12 (5.3)	1	1–1	1–1
Men	8 (3.2)	1	1–1	1–1	13 (5.3)	1	1–1	1–2
Cerebrovascular diseases (I60-I69)								
Total					19 (4.0)	1	1–1	1–1
Women					10 (4.4)	1	1–1	1–1
Men					9 (3.6)	1	1–1	1–1
Acute upper respiratory infections(J00-J06)								
Total					16 (3.4)	1	1–1	1–3
Women					6 (2.7)	1	1–1	1–1
Men					10 (4.0)	1	1–1	1–3
Influenza and pneumonia (J09-J18)								
Total	10 (2.1)	1	1–1	1–2	118 (25.0)	1	1–2	1–5
Women	4 (1.8)	1	1–2	1–2	41 (18.2)	1	1–2	1–5
Men	6 (2.4)	1	1–1	1–1	77 (31.2)	1	1–2	1–4
Other acute lower respiratory infections (J20-J22)								
Total					14 (3.0)	1	1–1	1–2
Women					9 (4.0)	1	1–2	1–2
Men					5 (2.0)	1	1–1	1–1
Lung diseases due to external agents (J60-J70)								
Total					13 (2.8)	1	1–1	1–2
Women					6 (2.7)	1	1–1	1–2
Men					7 (2.8)	1	1–1	1–1
Diseases of oesophagus, stomach and duodenum (K20-K31)								
Total	19 (4.0)	1	1–1	1–2	21 (4.4)	1	1–1	1–6
Women	8 (3.6)	1	1–1	1–1	11 (4.9)	1	1–1	1–6
Men	11 (4.5)	1	1–1	1–2	10 (4.0)	1	1–1	1–5
Hernia (K40-K46)								
Total	29 (6.1)	1	1–2	1–2	12 (2.5)	1	1–1	1–1
Women	6 (2.7)	1	1–2	1–2	1 (0.4)	1	1–1	1–1
Men	23 (9.3)	1	1–2	1–2	11 (4.5)	1	1–1	1–1
Noninfective enteritis and colitis (K50-K52)								
Total	11 (2.3)	1	1–2	1–6				
Women	8 (3.6)	1	1–3	1–6				
Men	3 (1.2)	2	2–2	2–2				
Other diseases of intestines (K55-K63)								
Total	22 (4.7)	1	1–2	1–5	30 (6.4)	1	1–2	1–9
Women	16 (7.1)	1	1–2	1–4	17 (7.6)	1	1–2	1–9
Men	6 (2.4)	1	1–2	1–5	13 (5.3)	1	1–1	1–3
Disorders of gallbladder, biliary tract and pancreas (K80-K87)								
Total					12 (2.5)	1	1–1	1–3
Women					5 (2.2)	1	1–1	1–3
Men					7 (2.8)	1	1–1	1–3
Other diseases of the digestive system (K90-K93)								
Total					19 (4.0)	1	1–1	1–3
Women					6 (2.7)	1	1–1	1–2
Men					13 (5.3)	1	1–1	1–3
Arthropathies (M00-M25)								
Total	56 (11.9)	2	2	6	27 (5.7)	1	1	3
Women	37 (16.4)	2	2	5	15 (6.7)	1	1	3
Men	19 (7.7)	1	2	6	12 (4.9)	1	1	2
Soft tissue disorders related to use, overuse and pressure (M70-M79)								
Total					29 (6.1)	1	1–1	1–2
Women					15 (6.7)	1	1–1	1–2
Men					14 (5.7)	1	1–1	1–2
Disorders of bone density and structure (M80-M85)								
Total	11 (2.3)	2	1–2	1–3				
Women	6 (2.7)	2	1–2	1–3				
Men	5 (2.0)	2	2–2	1–2				
Renal tubulo-interstitial diseases (N10-N16)								
Total					11 (2.3)	1	1–1	1–2
Women					4 (1.8)	1	1–1	1–1
Men					7 (2.8)	1	1–1	1–2
Other diseases of urinary system (N30-N39)								
Total					39 (8.3)	1	1–1	1–6
Women					20 (8.9)	1	1–2	1–6
Men					19 (7.7)	1	1–1	1–2
Non-inflammatory disorders of female genital tract (N80-N98)								
Total	12 (2.5)	1	1–1	1–1				
Women	12 (5.3)	1	1–1	1–1				
Men	0 (0)							
Congenital malformations of the circulatory system (Q20-Q28)								
Total	10 (2.1)	1	1–2	1–5				
Women	5 (2.2)	1	1–1	1–2				
Men	5 (2.0)	2	1–2	1–5				
Chromosomal abnormalities, not elsewhere classified (Q90-Q99)								
Total	108 (22.9)	1	1–1	1–3	33 (7.0)	1	1–1	1–3
Women	53 (23.6)	1	1–1	1–3	19 (8.4)	1	1–1	1–2
Men	55 (22.3)	1	1–1	1–3	14 (5.7)	1	1–1	1–3
Symptoms and signs involving the circulatory and respiratory systems (R00-R09)								
Total					29 (6.1)	1	1–1	1–7
Women					8 (3.6)	2	1–3	1–7
Men					21 (8.5)	1	1–1	1–3
Symptoms and signs involving the digestive system and abdomen (R10-R19)								
Total	18 (3.8)	1	1–1	1–4	64 (13.6)	1	1–1	1–25
Women	12 (5.3)	1	1–2	1–4	35 (15.6)	1	1–2	1–25
Men	6 (2.4)	1	1–1	1–1	29 (11.7)	1	1–1	1–2
General symptoms and signs (R50- R69)								
Total					68 (14.4)	1	1–1	1–5
Women					38 (16.9)	1	1–1	1–5
Men					30 (12.1)	1	1–1	1–2
Injuries to the head (S00-S09)								
Total					29 (6.1)	1	1–1	1–3
Women					19 (8.4)	1	1–1	1–3
Men					10 (4.0)	1	1–1	1–3
Injuries to abdomen, lower back, lumbar spine and pelvis (S30-S39)								
Total					10 (2.1)	1	1–1	1–2
Women					7 (3.1)	1	1–2	1–2
Men					3 (1.2)	1	1–1	1–1
Injuries to the shoulder and upper arm (S40-S49)								
Total	10 (2.1)	1	1–1	1–2	15 (3.2)	1	1–1	1–2
Women	7 (3.1)	1	1–1	1–2	10 (4.4)	1	1–1	1–1
Men	3 (1.2)	1	1–1	1–1	5 (2.0)	1	1–1	1–2
Injuries to the elbow and forearm (S50-S59)								
Total					14 (3.0)	1	1–1	1–2
Women					7 (3.1)	1	1–2	1–2
Men					7 (2.8)	1	1–1	1–1
Injuries to the hip and thigh (S70-S79)								
Total	16 (3.4)	1	1–2	1–2	53 (11.2)	1	1–2	1–3
Women	12 (5.3)	1	1–2	1–2	26 (11.6)	1	1–2	1–3
Men	4 (1.6)	1	1–1	1–1	27 (10.9)	1	1–1	1–2
Injuries to the knee and lower leg (S80-S89)								
Total	23 (4.9)	1	1–1	1–2	39 (8.3)	1	1–1	1–4
Women	13 (5.8)	1	1–1	1–2	28 (12.4)	1	1–1	1–4
Men	10 (4.0)	1	1–1	1–2	11 (4.5)	1	1–2	1–3
Injuries to the ankle and foot (S90-S99)								
Total					20 (4.2)	1	1–1	1–1
Women					9 (4.0)	1	1–1	1–1
Men					11 (4.5)	1	1–1	1–1
Effects of foreign body entering through natural orifice (T15-T19)								
Total					24 (5.1)	1	1–1	1–2
Women					7 (3.1)	1	1–1	1–2
Men					17 (6.9)	1	1–1	1–2
Health services for examination and investigation (Z00-Z13)								
Total	62 (13.1)	1	1–1	1–3	19 (4.0)	1	1–1	1–3
Women	36 (16.0)	1	1–1	1–3	6 (2.7)	1	1–2	1–3
Men	26 (10.5)	1	1–1	1–2	13 (5.3)	1	1–1	1–1
Health services for specific proce-dures and health care (Z40-Z54)								
Total	12 (2.5)	1	1–1	1–6				
Women	5 (2.2)	1	1–1	1–1				
Men	7 (2.8)	1	1–1	1–6				
Persons with potential health hazards related to family and personal history and certain conditions influencing health status (Z80-Z99)								
Total	17 (3.6)	1	1–1	1–4				
Women	10 (4.4)	1	1–2	1–4				
Men	7 (2.8)	1	1–1	1–1				
